# Head and Neck Positions Affect Equine Kinematic Variables in Marcha Batida Gait—A Pilot Study

**DOI:** 10.3390/ani15081090

**Published:** 2025-04-09

**Authors:** Natália Almeida Martins, Brunna Patrícia Almeida Fonseca, Amanda Piaia Silvatti, Fabrício Luciani Valente, Nara Luisa Soares, Samuel Pereira Simonato, Laura Patterson Rosa, Millena Oliveira Andrade, Kate Moura da Costa Barcelos

**Affiliations:** 1 Departamento de Veterinária, Universidade Federal de Viçosa, Viçosa 36570-900, MG, Brazil; natalia.am25@gmail.com (N.A.M.); fabriciovalente@ufv.br (F.L.V.); 2 Independent Researcher, Sao Paulo 04548-902, SP, Brazil; brunnapatricia@gmail.com; 3 Departamento de Educação Física, Universidade Federal de Viçosa, Viçosa 36570-900, MG, Brazil; amandasilvatti@ufv.br (A.P.S.); nara.soares@ufv.br (N.L.S.); 4 Medicina Veterinária, Centro Universitário de Itajubá—FEPI, Itajubá 37501-002, MG, Brazil; simonatovet@gmail.com; 5 Department of Veterinary Clinical Sciences, Lewyt College of Veterinary Medicine, Long Island University, Brookville, NY 11548, USA; laura.dasnevespattersonrosa@liu.edu; 6 Departamento de Zootecnia, Escola de Medicina Veterinária e Zootecnia, Universidade Federal de Goiás, Goiânia 05508-270, GO, Brazil; millena.o.a@gmail.com

**Keywords:** stride, dissociation, gaited horses, Mangalarga Marchador

## Abstract

The Mangalarga Marchador (MM) breed naturally performs four-beat gaits known as “Marcha”. Kinematic analysis can provide insights for optimizing training and competition performance in this breed while potentially mitigating welfare concerns associated with extreme head and neck positions (HNPs) applied without an adequate understanding of their impacts. We examined how different HNPs affect the MM horse’s Marcha Batida gait. Four HNPs were evaluated: HPN1—loose reins, HPN2—the competition standard for MM, HPN3—an extremely elevated head and neck, and HPN4—a slightly behind-the-vertical position. Kinematic data were collected using an optoelectronic system, and diagonal dissociation, stride length, and step height were analyzed. HNP3 had the highest dissociation, shortest stride length, and higher step height compared to the other HNPs. HNP1 resulted in the longest stride length. HNP2 and HNP4 differed in diagonal dissociation. HNPs significantly impact the kinematics of the Marcha Batida gait in this cohort of Mangalarga Marchador horses. Our results confirm the benefits of HNP2, the recommended position for shows and competitions. Additionally, it discourages the use of HNP3 and reiterates the need for further research into HNPs in four-beat gaits, highlighting the importance of rider training and the careful selection of HNPs to optimize Marcha Batida performance.

## Simple Summary

The head and neck position (HNP) of a horse while ridden may affect the trot movement, yet in four-beat gaits such as the Marcha Batida, there is little understanding of the impact of HNP despite current riding and training recommendations for specific positions. We evaluated four distinct HNPs and their association with diagonal dissociation, stride length, and step height as these are key factors in the Marcha Batida. Nine Mangalarga Marchador horses used for leisure and naturally performing the Marcha Batida gait were included in this evaluation. The HNP3 resulted in the highest diagonal dissociation yet negatively affected stride and step parameters. HNP4 also had an overall undesired performance in gait parameters within this group of horses. HNP2, a position aligned with welfare best practices, had the best overall parameters with significantly different diagonal dissociation, reiterating the benefits of this position for the Marcha Batida. We demonstrate that spatial coordinate data can be used for evaluating head and neck positions and their effects on stride length, step height, and diagonal dissociation in Marcha Batida.

## 1. Introduction

Kinematic analysis is used in sports veterinary medicine to better understand equine movement, further supporting equestrian sports [1,2,3,4]. In horses that naturally perform the trot (a diagonally coupled locomotion pattern intercalated by moments of suspension), head and neck position affect limb angle, support time, and demonstrate a diverse effect on posture when walking [5] and trotting [6], suggesting that riders may alter the locomotion pattern through riding aids and that this can possibly impact equine competition performance. While biomechanical characteristics of trot and their relation with equitation [7] demonstrate a relationship between head and neck position with biomechanical changes [6,8,9], there is a lack of research into these aspects in four-beat gaited breeds in special Brazilian-native horses such as the Mangalarga Marchador. Four-beat gaits can be characterized by the absence of a suspension phase and tripodal support stances intercalating diagonal and lateral stances [10,11,12].

The Mangalarga Marchador (MM) is one of the most common equine breeds in Brazil, with over 600 thousand registered individuals [13]. The breed is popular for its four-beat gaits, classified as Marcha Batida (predominantly diagonal stances) and Marcha Picada (predominantly lateral stances) [11,13,14,15]. Still, the breed standard and ideal gait parameters are subjective and not quantitatively described. As translated from the breed standard: “Marcha batida/picada is a natural, symmetrical, four-beat gait with alternating lateral and diagonal leg stances, intercalated by triple support moments. Ideally, it’s a regular movement, elastic, with overreaching hindlimbs, balanced, subtle front leg semicircular motion, and good joint flexibility” [16]. As described by the MM Regulations for breed judges, a long, symmetrical (between diagonal pairs) step length and a medium height on forelimb steps are also desirable [17]. Although kinematic analysis has been used to describe the Marcha Batida [2,9,11,15,18,19,20,21], MM breeders and trainers also believe diagonal dissociation to be a major contributing factor in gait quality and rider comfort. Diagonal dissociation refers to the subtle timing differences in the landing phase between diagonal limbs [22]. In horses performing the trot, a hindlimb-first or “positive” diagonal dissociation is preferred for maintaining trunk pitch stability and reducing mechanical energy losses [22], yet this aspect has not been evaluated in MM gaits. Thus, the lack of quantitative parameters regarding diagonal dissociation and possible association to riding head and neck position warrant further research, given the perceived importance of these aspects and diverse training methodologies employed to optimize them.

Our goal is to evaluate the diagonal dissociation and other kinematic alterations in a small MM horse group that naturally performs Marcha Batida, subjected to four different head and neck positions previously described [6]. We hypothesize that head and neck positions may influence diagonal dissociation, step height, and stride length for the Marcha Batida gait. The results from this pilot study provide a preliminary understanding of rider effect in diagonal dissociation and support further research into suitable head and neck positions that also contribute to animal welfare and more ethical riding.

## 2. Materials and Methods

### 2.1. Animals

Nine MM horses (4 mares and 5 geldings) were enrolled in this study, with a mean weight of 343 kg (SD ± 29.3), mean height of 1.49 m (SD ± 0.04 m), and aged between 4 and 12 years old (mean = 7.11, SD ± 2.52). All animals were used for leisure and low-level athletic activity. The horses used in this study were deemed healthy and clinically examined for lameness according to Stashak [23] and Fonseca’s [24] back pain protocols both in hand and ridden. All the horses in this cohort naturally perform the Marcha Batida gait and are ridden most commonly in a free or natural head and neck position, decreasing the chances of a possible competition training bias.

### 2.2. Kinematic Data Acquisition

**Figure 1:**
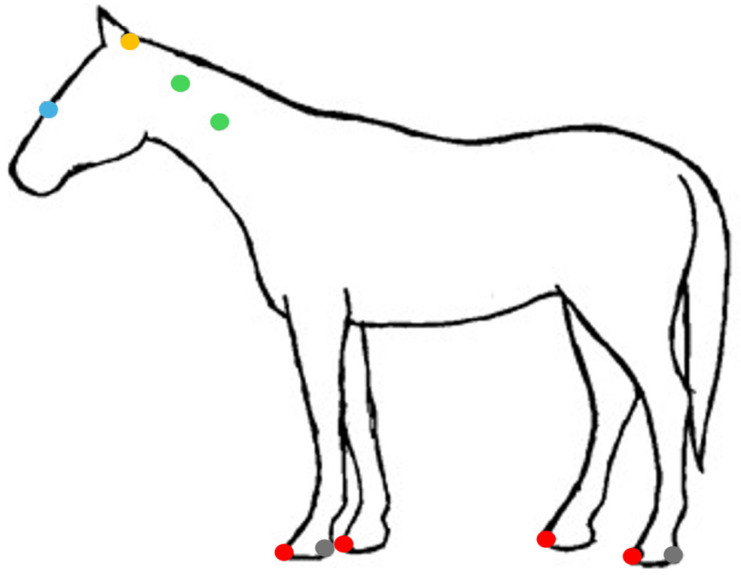
Marker placement in the anatomical landmarks of interest of the horse body and hooves, where blue = nasoincisive notch, yellow = poll, green = transverse processes of cervical vertebrae 3 and 5, gray = lateral heel, and red = central point of the toe.

Markers were attached using hypoallergenic double-sided tape in the following anatomical landmarks: nasoincisive notch, poll, cervical vertebrae 3 and 5 transverse processes, lateral heel, and in the central point of the toe of all 4 hooves, identified by palpation of the underlying skeletal structures, and placed by a single person (N.A.M.) (Figure 1).

**Figure 2:**
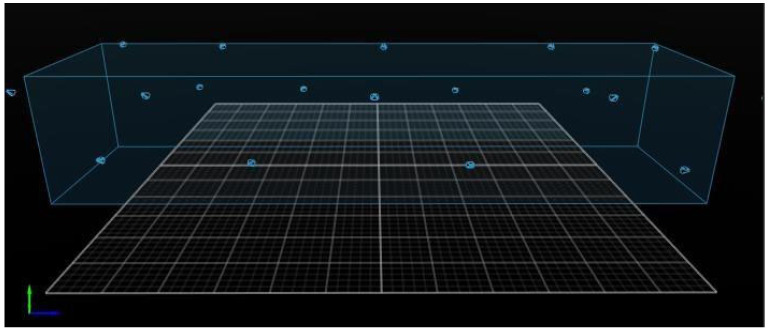
Visual demonstration of the acquisition field and location of each camera connected to the tracking system in the capture field on Motive MTV-BDY2 OptiTrack.

Nineteen optoelectronic OptiTrack Prime 17W (NaturalPoint, Inc., Corvallis, OR, USA) (360 Hz) cameras were positioned in a hangar at the alternating height of 3 m and 1.5 m connected to Motive MTV-BDY2 OptiTrack (NaturalPoint, Inc., Corvallis, OR, USA), allowing all markers to be captured simultaneously. The acquisition field total volume was 16 × 4.8 × 3 m, corresponding to the length, width, and height (Figure 2).

**Figure 3:**
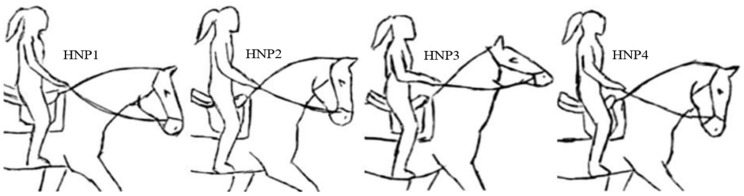
Visual representation of the evaluated head and neck positions (HNPs) of 9 horses ridden by a single rider: HNP1 = free or natural. HNP2 = neck raised, poll high, and bridge of the nose slightly in front of the vertical axis, the competition MM reference position. HNP3 = neck extremely elevated and the bridge of the nose considerably in front of the vertical. HNP4 = neck raised, poll high, and bridge of the nose slightly behind the vertical (modified from Rodhin [6]).

A single professional rider (height = 1.63 m, 77 kg) rode each individual horse in Marcha Batida with a mean speed 3.48 (±0.14) ms^−1^ through the acquisition field for each of the head and neck positions (HNPs). The different head and neck positions were achieved through reins and body posture cues (Figure 3), as described: HNP1 = free or natural, a voluntarily acquired position, unrestrained with loose reins; HNP2 = neck raised, poll high, and bridge of the nose slightly in front of the vertical axis, the competition MM reference position; HNP3 = neck extremely elevated and the bridge of the nose considerably in front of the vertical; and HNP4 = neck raised, poll high, and bridge of the nose slightly behind the vertical (modified from Rhodin [6]). All individuals in this study are normally ridden in HPN1 or HPN2 as per information of the owners and trainers. For each horse, the order of each HNP was randomly assigned using a random number picker tool (Google.com) representing each HNP, and each horse was worked in the four HNPs in a single day. The positions were evaluated by a Brazilian Association of Mangalarga Marchador Horse Breeders (ABCCMM) registered and trained official inspector (K.M.C.B.).

Riding equipment comprised the saddle and its attachments, saddle pad, and bit/bridle normally utilized and most comfortable or accepted by each horse. Equipment was checked for fit by the investigators prior to each ridden assessment, and no fitting issues were noted in this cohort.

Horses were ridden for 5 min before the data collection to warm up and be exposed to the recording environment. All horses were subjected to each HNP for about 1–4 min prior to data collection in order to adapt and maintain the HNP through the camera acquisition field. The data were captured in triplicate for each HNP. Each capture had at least 5 complete gait cycles (e.g., a support stance and its next recurrence), as recommended by Clayton and Schamhardt [25]. Each horse had an interval of 2–5 min between data collection for each HNP, allowing for the adaptation and 1 min of rest between passes, depending on individual fitness and willingness to maintain the HNP.

This approach allowed for a total of 15 complete gait cycles per horse, per HNP, resulting in 135 complete gait cycles or 143 diagonal dissociation observations per HNP. Horses were ridden for a maximum of 100 min or 1 h and 40 min for each horse, less than the leisure/trail rides these individuals were commonly subjected to according to their exercise schedule.

### 2.3. Statistical Methods

**Figure 4:**
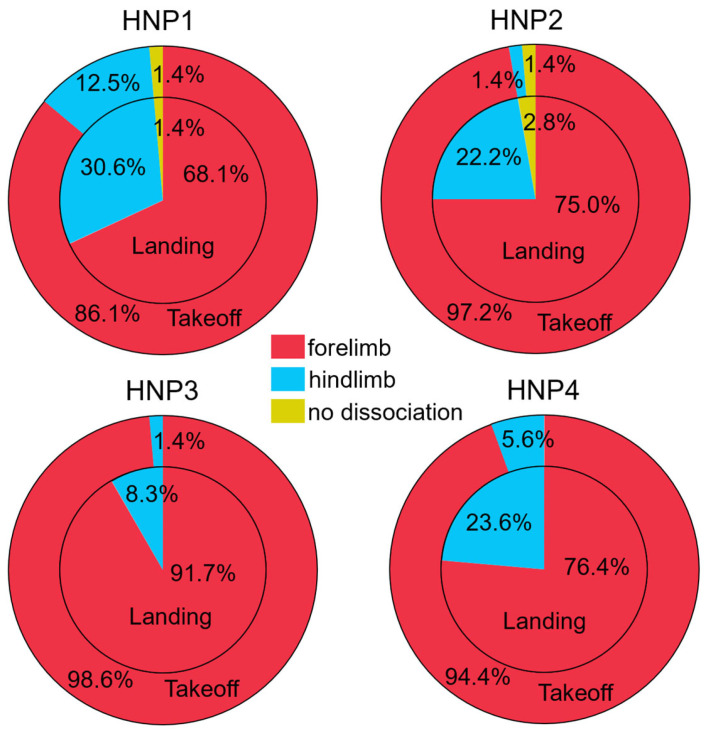
Percentage of forelimb-first (red), hindlimb-first (blue), or no dissociation (yellow) observed by each HNP, where the inner pie graph represents landing and the outer pie graph represents takeoff. The effects of HNPs were evaluated in 9 horses ridden by a single rider. Intra-individual variation is not represented, yet it is accounted for in the statistical model.

To reduce signal errors, spatial coordinate data obtained in Motive MTV-BDY2 were filtered using the Butterworth filter, as recommended [26]. We obtained diagonal dissociation as a percentage, calculated based on the following: frame difference between the front or diagonal opposite limb contact time (either landing or takeoff) divided by the respective complete gait cycle time [11]. The percentage of takeoff dissociation was calculated based on the moment the hoof leaves the ground, while the landing dissociation percentage corresponds to when the hoof touches the ground [11]. For each dissociation, the leading limb was reflected as positive dissociation values for the hindlimb, and negative values for the forelimb first, and was predominantly represented in all HNPs with forelimb-first values (Figure 4). As breed standards do not dictate which type of dissociation is preferred (hindlimb versus forelimb) and negative dissociation values are not physiologically possible, we aimed to evaluate the diagonal dissociation percentage regardless of the leading limb by transforming data to positive values for statistical analysis. A Restricted Maximum Likelihood (REML) linear mixed model for repeated measures (individual horse) was performed on JMP Pro Version 16.0 (SAS Institute Inc., Cary, NC, USA), where the reference limb and type of dissociation (takeoff or landing) were fixed effects, and each HNP a variable.

Step length was obtained by the difference between each limb landing frame minus the following frame when the same limb lands again. As step length represents the difference between each limb landing frame minus the following frame when the same limb lands again, we evaluated if there was a difference in step length related to forelimb versus hindlimb within each diagonal pair and calculated the overall stride length for each gait cycle based on the mean value between all step lengths. Step height (highest point achieved in the vertical plane by both the heel and toe markers per stride cycle) was averaged by the hindlimb and forelimb per individual horse and HNP. The step length, stride length, and step height were evaluated for normal distribution using Anderson–Darling goodness of fit and subjected to a 2-way repeated measures ANOVA considering the interaction of the HNP and reference limb on JMP Pro Version 16.0 (SAS Institute Inc., Cary, NC, USA). To account for multiple comparisons, the significance threshold was corrected using Bonferroni’s correction, where there was a *p* of 0.05 divided by 7 tests, resulting in a significance threshold of *p* ≤ 0.0071. Tukey’s honestly significant difference (HSD) test was used to compare the means of significant ANOVA tests.

## 3. Results

### 3.1. Diagonal Dissociation

**Figure 5:**
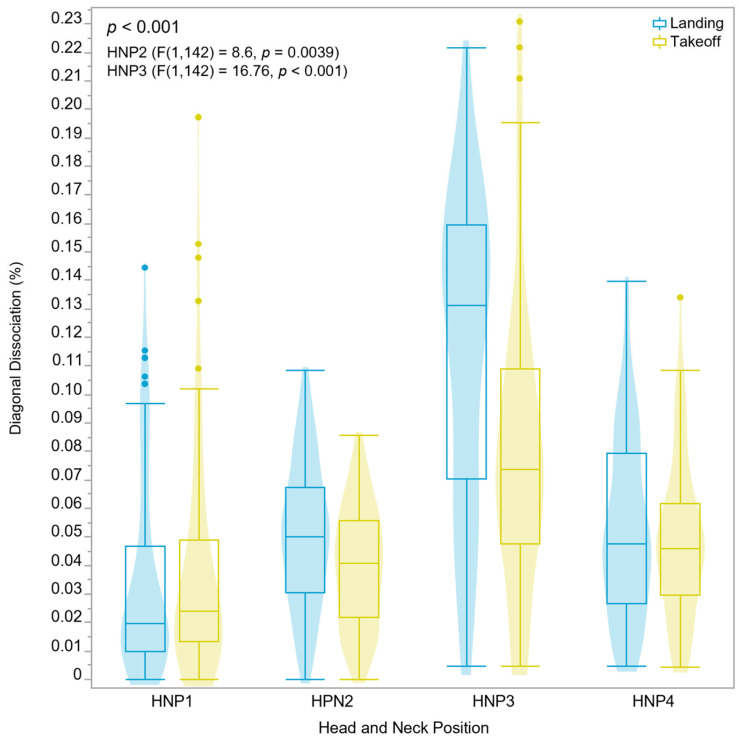
Diagonal dissociation values by each HNP and type of dissociation (landing = blue, takeoff = yellow) represented by violin and boxplot, and outliers represented by individual circles. The diagonal dissociation means were significantly different for HNP2 and HNP3. The effects of HNPs were evaluated in 9 horses ridden by a single rider. Intra-individual variation is not represented, yet it is accounted for in the statistical model.

The diagonal dissociation percentage for both takeoff and landing was significantly influenced by HNP2 and HNP3 (*p* < 0.001). We also observed a higher mean dissociation percentage (takeoff = 0.08%, SD ± 0.05%; landing = 0.12%, SD ± 0.05%) for HNP3 (F(1,142) = 16.76, *p* < 0.001) and smaller but significantly different dissociation percentages for HNP2 (F(1,142) = 8.6, *p* = 0.0039), with the landing dissociation values (mean = 0.05%, SD ± 0.03%) higher than the takeoff dissociation (mean = 0.04%, SD ± 0.02%) (Figure 5). HNP1 (landing mean = 0.05%, SD ± 0.03%; takeoff mean = 0.04%, SD ± 0.04%) and HNP4 (landing mean = 0.07%, SD ± 0.10%; takeoff mean = 0.07%, SD ± 0.10%) were not statistically different in terms of diagonal dissociation (%).

### 3.2. Step and Stride Length

**Figure 6:**
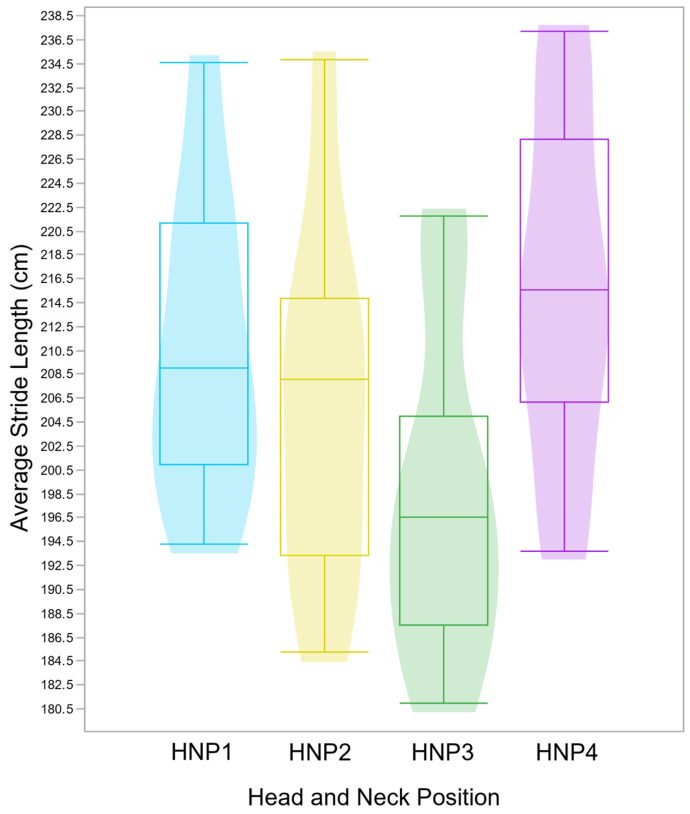
Boxplots and violin plots demonstrate the stride length distribution per HNP, where HNP1 (blue) and HPN4 (purple) have a significantly (*p* = 0.0026) longer mean stride length in cm than HNP3 (green), while the HNP2 (yellow) mean is statistically similar to all others. The effects of HNPs were evaluated in 9 horses ridden by a single rider. Intra-individual variation is not represented, yet it is accounted for in the statistical model.

Step length had no statistical difference between forelimb and hindlimb within the diagonal pair and between diagonal pairs (*p* = 0.9, Supplementary Table S1), demonstrating that the step length is statistically similar between the limbs composing the diagonal limb pairs in this cohort. Stride length differed between HNPs (F(3,68) = 5.25, *p* = 0.0026), where HNP1 (mean = 210.3 cm, SD ± 12.7cm) and HNP4 (mean = 215.9 cm, SD ± 14.6cm) were, respectively, 12.3 cm and 17.9 cm longer on average than HNP3 (mean = 198.0 cm, SD ± 13.6cm), and HNP2 (mean = 206.0 cm, SD ± 14.8 cm) was statistically similar to the other positions (Figure 6).

### 3.3. Step Height

**Figure 7:**
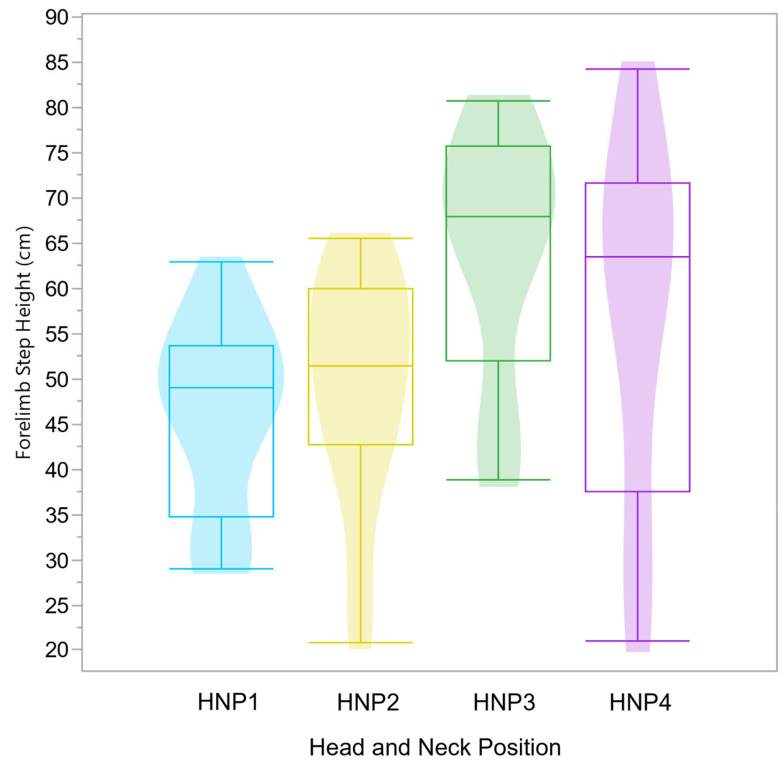
Boxplots and violin plots demonstrate the forelimb step height distribution per HNP, where HNP1 (blue) and HPN2 (yellow) have a significantly (*p* = 0.0058) lower vertical peak mean height in cm than HNP3 (green), while the HNP4 (purple) mean is statistically similar to all others. The effects of HNPs were evaluated in 9 horses ridden by a single rider. Intra-individual variation is not represented, yet it is accounted for in the statistical model.

While hindlimb step height was not significantly different between HNPs (F(3,68) = 3.74, *p* = 0.015), HNP had a significant effect on forelimb step height (F(3,68) = 5.37, *p* = 0.0022). HNP3 resulted in the highest forelimb mean step height with 65.6 cm (SD ± 13.9 cm), 14.1 cm higher than HNP2 (mean = 51.5 cm, SD ± 13.7 cm) and 18.2 cm higher than HNP1 (mean = 47.4 cm, SD ± 10.4 cm) (Figure 7). HNP4 forelimb step height was not significantly different from the other HNPs (mean = 58.8 cm, SD ± 19.6 cm) (Supplementary Table S2). HNP1 resulted in the lowest forelimb mean step height for this cohort.

## 4. Discussion

Our results demonstrate that head and neck positions may influence diagonal dissociation, step height, and stride length for the Marcha Batida gait. While the percentage of dissociation and footfall sequence is the baseline for the current Marcha standards [9], historically, training protocols for Marcha gaited horses have been based on research conducted in breeds that naturally perform the trot. In sport horses, the head and neck position (HNP) in which the horses are ridden are considered crucial aspects of animal welfare [27]. HPN influences behavior, cortisol levels, heart rate [28], trachea diameter [29], intrathoracic pressure [30], and the flexion–extension of the thoracolumbosacral [5], impacting both the performance and well-being of the horse. Riders can influence head and neck position through riding cues, equipment, and training, altering the limb amplitude (height and length achieved) of movement [31]. Furthermore, while young horses and horses with limited training may be more affected by changes in HNP than high-performance horses, HNP still significantly influences the kinematics of equine trot [6].

Despite the graphical distribution of the kinematic parameters perceived as different for some HNPs, these are visual representations of the data and should not be overinterpreted, as the REML model utilized for the analysis accounts for individual repeated measures, which is not represented in the distribution graphs. Furthermore, the number of horses (N = 9) in this study is similar or higher than other published research in HNPs, and the number of gait cycles increases the number of total observations per trait, improving the statistical analysis power [1,6,7,8,26]. Interestingly, we observed a prevalence in forelimb-first (negative dissociation) for both landing and takeoff dissociation in all HNPs (Figure 4). In horses performing the trot, hindlimb-first diagonal dissociation during landing is preferred [22]; thus, the effect of the leading limb needs to be further studied in the Marcha Batida.

Of the six originally described HPNs [6], the selected four included in our study are the more commonly observed in MM leisure riding, competitions, and training, in the authors’ professional experience with the breed. While the four HNPs are described for trotting horses [6], their effects on the MM Marcha Batida still require further evaluation. In particular, HNP1 is considered to be a free or natural position for the horse and HNP2 has been previously recommended by the Fédération Equestre Internationale (FEI) for sport horses in training and competition [32,33]. HNP1, a position described as “free” or natural, demonstrated no significant difference between diagonal dissociation percentages of takeoff and landing and the lowest mean step height. The longer stride length in HNP1 can be compared to high-performance Warmblood dressage horses that at a walk demonstrate maximum stride amplitude in HNP1 [34]. This HNP also resulted in the highest percentage of hindlimb-leading landing dissociations. These observations could be due to the more natural head and neck position allowing for increased limb horizontal extension, with decreased vertical movement. HPN1 also caused the least diagonal dissociation percentage and the lowest quantitative mean step height, subjectively evaluated as undesirable traits in Marcha Batida.

Akin to observations for the trot [6,32], HNP2 is speculatively considered by the MM breeder association and judges as the ideal position for competition, regardless of gait type and stride length variation, collected to extended [35,36]. In HNP2, there was an increased takeoff and landing diagonal dissociation, average stride length, and lower/intermediate step height, suggesting that this position can be utilized in riding the Marcha Batida for moderate outcomes while improving diagonal dissociation. Dissociation values achieved by HNP2 can be explained by an improved vertebral column flexion–extension [34], which is desirable in the Marcha Batida. Furthermore, step height is an important aspect in the MM competition, and excessive or exaggerated movement can be penalized by the judges, as it can be implicated in increased limb and joint concussion and damage [6,8,32]. Changes in limb mass distribution due to dissociation can help reduce energy loss during hoof contact [22], potentially preventing lesions.

Although the diagonal dissociation percentage in landing and step height was the highest in HNP3, the stride length for this HNP was significantly smaller when compared to HNP1 and HNP4 and had no statistical difference compared to HNP2. HNP3 is considered a “high-set and stressed position” due to its effects on behavior and locomotor apparatus [6,8,34]. It also caused the lowest percentage of hindlimb-first diagonal dissociation in the landing of all HNPs. This head and neck position is associated with negative health consequences due to its extreme height and the stress it induces [37]. The observed shorter stride length is potentially caused by the reduced thoracolumbosacral range of motion [32] restricting limb pro-retraction, consistent with the findings in other breeds [6,8,32,35,38]. In the trot, extremely elevated HNPs can predispose the horse to back injuries, especially if the horse is required to maintain the HNP3 for prolonged times [6]. Despite its desirable outcomes regarding diagonal dissociation, the high-set head and neck, leading to a stressed epaxial musculature with an overextended vertebral column in HNP3, suggests that this HNP may be detrimental to horse welfare. The extreme step height along with shorter stride length are also both undesired traits according to the MM breed standard [17]. The observed kinematic changes for HNP3 further discourage its use in MM Marcha Batida horses.

HNP4, maintaining the nose slightly behind the vertical [32], resulted in the second-highest step height, hindlimb-first landing dissociation, as well as the longest stride length. The desirable aspect of the longest stride length, as well as second highest step height and second highest percentage of hindlimb-first during landing, may be of interest to the Marcha Batida during competition [17], as a behind-the-vertical position has been associated with higher performance marks in dressage horses [39]. Still, this position is also negatively implicated in animal welfare and has been associated with conflict behaviors, stronger startle reactions, increased anxiousness, as well as excessive stretching of the nuchal ligament [39]. While HNP4 is a potential position during specific training moments, according to the current FEI guidelines [32,33], the nose behind the vertical is discouraged for extended periods of time due to its possible behavioral and health implications. Still, the tested HNP4 in this cohort was less than <10° behind the vertical, which may be different than the positions tested in previous research [39]; thus, a further evaluation of the potential effects of this position is recommended.

While our results may support further research into the HNPs and their influences in the four-beat Marcha gaits of Brazilian-native horses, our pilot study is also limited by a small, leisure riding population of a single gait type. Further research evaluating larger populations, including actively competing and high-level performance MM horses, as well as individuals of Marcha Picada, is recommended to substantiate our preliminary findings and develop ideal riding and showing HPNs for the breed and gait type. The long-term effects of HNPs, as well as individual rider effects, should also be evaluated in future efforts. Nonetheless, it is crucial to recognize that monitoring the head and neck position can be an effective way to implement equine welfare practices and contribute to an ethic and effective training in four-beat gaited breeds.

## 5. Conclusions

Head and neck positions can be altered by the rider during the Marcha through reins and body posture and influence equine locomotion kinematic variables, suggesting that rider cues and HNP influence the dissociation, stride length, and stride height of Marcha Batida gait in Mangalarga Marchador horses. While the diagonal dissociation percentage, a trait desired by the breed, was higher in HNP3, it also resulted in the shortest mean stride length and higher stride height, both characteristics that are undesired according to breed standards. Furthermore, the use of this position in horse training is not recommended due to the possible negative impact on animal welfare demonstrated in other studies [6,8,34]. As the recommended position for MM horses, the HNP2 results reiterate the possible benefits of this position in the Marcha Batida. Our pilot results support the evaluation of HNP effects utilizing spatial coordinate data in four-beat gaited breeds.

## Data Availability

Supplementary data associated with this article can be found, in the online version, at “OSF” in https://osf.io/rnz68/. doi:10.17605/OSF.IO/RNZ68.

## Supplementary Materials

The following supporting information can be downloaded at: https://www.mdpi.com/article/10.3390/ani15081090/s1. Table S1. Head and neck position (HNP) effect on the stride length (cm) per limb. Table S2. Head and neck position (HNP) effect on step height (cm) per limb.
