# Supplementary material for: Head and Neck Positions Affect Equine Kinematic Variables in Marcha Batida Gait—A Pilot Study
Source: Animals (Basel). 2025 Apr 9;15(8):1090. doi: 10.3390/ani15081090 (PMC12024456; doi:10.3390/ani15081090)
Supplement: Supplementary file 1 [file animals-15-01090-s001.zip › animals-3532170-supplementary.pdf]

Supplemental Table 1. Head and Neck Position (HNP) effect on the step length (cm) per limb.

HNP	Limb	Minimum	Maximum	Median	Mean	SD±
1	RP	205.28	236.53	228.97	225.32	11.81
1	LP	219.29	246.79	223.01	229.20	10.56
1	RT	211.36	242.5	223.24	225.90	09.98
1	LT	205.54	237.91	228.02	224.81	10.56
2	RP	169.68	239.73	202.4	199.57	20.82
2	LP	171.23	242.87	203.2	202.75	21.19
2	RT	157.85	238.23	200.3	197.01	21.71
2	LT	159.09	242.43	198.8	196.55	22.96
3	RP	161.39	215.4	179.47	184.55	17.71
3	LP	162.71	216.02	185.42	187.44	18.10
3	RT	168.98	209.57	183.11	187.90	14.73
3	LT	169.06	206.42	184.66	187.70	14.74
4	RP	186.65	216.93	200.77	201.93	10.57
4	LP	187.52	225.7	201.36	202.71	13.13
4	RT	180.02	227.49	189.45	196.32	15.01
4	LT	189.92	223.21	207.08	206.10	12.12

RP – right hindlimb; LP – left hindlimb; RT – right forelimb; LT – left forelimb; SD – Standard Deviation.

Supplemental Table 2. Head and Neck Position (HNP) effect on step height (cm) per limb.

HNP	Limb	Minimum	Maximum	Median	Mean	SD±
1	RP	19.55	63.09	39.65	42.45	13.26
1	LP	18.86	61.55	40.30	42.51	13.40
1	RT	31.75	70.21	53.17	52.53	10.58
1	LT	29.07	68.53	50.78	50.73	11.37
2	RP	28.97	63.96	43.17	45.24	12.20
2	LP	28.21	60.01	45.58	44.43	11.44
2	RT	37.35	69.85	56.88	55.81	10.27
2	LT	41.47	67.33	53.58	53.48	8.63
3	RP	27.00	76.78	57.16	55.50	16.06
3	LP	25.62	68.28	53.78	52.73	13.87
3	RT	41.55	79.36	69.01	65.44	12.80
3	LT	38.83	80.77	69.00	65.79	15.65
4	RP	22.90	71.64	56.92	52.11	16.53
4	LP	29.75	74.88	58.56	53.28	14.85
4	RT	31.63	84.18	65.45	62.02	16.36
4	LT	33.73	82.75	66.97	61.50	15.17

RP – right hindlimb; LP – left hindlimb; RT – right forelimb; LT – left forelimb; SD – Standard Deviation.
